# Numerical Calculation of High-Strength-Steel Saddle Plate Forming Suitable for Lightweight Construction of Ships

**DOI:** 10.3390/ma16103848

**Published:** 2023-05-19

**Authors:** Shun Wang, Jinliang Dai, Ji Wang, Rui Li, Jiayan Wang, Zhikang Xu

**Affiliations:** 1 Naval Architecture and Ocean Engineering College, Dalian Maritime University, Dalian 116026, China; daijinliang@dlmu.edu.cn (J.D.); wangjiayan@dlmu.edu.cn (J.W.); xuzhikang@dlmu.edu.cn (Z.X.); 2 School of Naval Architecture and Ocean Engineering, Dalian University of Technology, Dalian 116024, China; wangji@dlut.edu.cn (J.W.); lirui@dlut.edu.cn (R.L.)

**Keywords:** lightweight construction, plastic working, line heating, high-strength steel, plate forming

## Abstract

With the demand for construction of lightweight ships and polar ships, high-strength steel is increasingly applied in shipbuilding. There are a large number of complex curved plates to be processed in ship construction. The main method for forming a complex curved plate is line heating. A saddle plate is an important type of double-curved plate, which affects the resistance performance of the ship. The existing research on high-strength-steel saddle plates is lacking. To solve the problem of forming for high-strength-steel saddle plates, the numerical calculation of line heating for a EH36 steel saddle plate was studied. By combining it with a line heating experiment of low-carbon-steel saddle plates, the feasibility of numerical calculation based on the thermal elastic–plastic theory for high-strength-steel saddle plates was verified. Under the premise that the processing conditions such as the material parameters, heat transfer parameters, and the constraint mode of the plate were correctly designed, the effects of the influencing factors on deformation of the saddle plate could be studied by the numerical calculation method. The numerical calculation model of line heating for high-strength-steel saddle plates was established, and the effects of geometric parameters and forming parameters on shrinkage and deflection were studied. This research can provide ideas for the lightweight construction of ships and provide data support for automatic processing of curved plates. It can also provide inspiration for curved plate forming in fields such as aerospace manufacturing, the automotive industry, and architecture.

## 1. Introduction

The International Maritime Organization (IMO) has proposed new greenhouse gas emission reduction regulations, which were fully implemented in 2023 [1]. These regulations set higher requirements for the carbon emissions of vessels [2]. Carbon dioxide emissions of ships is the main pollution of the shipping industry [3]. Lightweight construction is a key way to reduce the carbon dioxide emissions of ships. Lightweight ships with more market competitiveness need to be constructed. With the development of the Arctic route, the safety of polar routes has become a hot topic of academic research. Ships and marine engineering equipment development are gradually moving towards the polar region. There are extremely high requirements of steel strength for polar ships, especially for ice breakers. Common ships face significant difficulties in navigating the Arctic region due to the challenges posed by low temperatures and collisions with floating ice [4]. The yield strength and tensile strength of high-strength steel are greater than that of low-carbon steel. The application of high-strength steel can not only meet the strength requirements of the hull, but also decrease the light weight to reduce the carbon emissions and improve the economic benefit. Apart from polar ships, high-strength steel is also increasingly used in container ships, liquefied natural gas (LNG) carriers, luxury cruise ships and offshore platforms. High-strength steel is an important material for manufacturing hull plates and it is significant to research the curved plate forming of high-strength steel [5]. Wang et al. [6] researched the bending strength of high-strength-steel rectangular profiles. For the forming process of high-strength-steel body panel, Guo et al. [7] optimized the process parameters in the drawing process. Dong et al. [8] conducted experiments to research the mechanical properties of DP980 steel plate laser forming under different laser parameters. Through the underwater welding test of high-strength steel, Fydrych et al. [9] proved that steel S355J2G3 and S500M are easy to crack under wet welding conditions and proposed a tempering bead technique to improve the wet weldability. To solve the problem of edge cracks generated during the stamping process of advanced high-strength steel, Jeong et al. [10] established a hole expansion ratio prediction model based on experiments. Kim et al. [11] evaluated the yield strength and tensile strength of high-manganese steel applied the fuel tanks in the ship powered by LNG after fiber laser welding.

There are a large number of complex curved plates to be processed in ship construction. A saddle plate is an important type of double-curved plates in ships. The primary method for forming hull curved plates is line heating [12], a plastic working process that allows for dieless forming without size limitations. This makes line heating a convenient and cost-effective solution for the formation of curved plates in ships. However, the automation degree of line heating is not high [13]. To solve the challenges in hull curved plate formation, scholars from different countries have performed a lot of research on line heating. Based on the experiment and numerical simulation of the line heating for single-curved plates, Seong et al. [14] proposed a method for calculating forming parameters from the geometric parameters of plates. Qi et al. [15] proved that the hydrogen and oxygen flame is used as the heat source of the line heating. Through experiments and numerical simulations, it has been proven that hydrogen and oxygen flames are more economical and efficient than acetylene flames. Kim et al. [16] proposed a classification method for hull curved plates based on convolutional neural networks in order to select the hot forming process of hull curved plates more efficiently. In order to achieve automation of the line heating process, Shibahara et al. [17] proposed an artificial intelligence line heating system by combining reinforcement learning and finite element method simulations. This system can deal with the nonlinear phenomena in line heating. For the line heating of low-carbon steel with a plate thickness of 0.02 m, Han et al. [18] proved that the coupling effect between the heating lines can be ignored when the heating line spacing is greater than 0.15 m. Yona et al. [19] designed the experiment and numerical calculation of line heating forming. The results of the two methods showed that the thermal deformation of the steel plate mainly depends on the plate thickness. For the induction heating of the steel plate, Chang et al. [20] studied the effect of curvature on the heat source distribution in line heating. Wang et al. [21] established a numerical calculation model of single-heating line for the forming of high-strength-steel sail plates and studied the effects of plate geometric and forming parameters on deformation. Based on the numerical calculation model of single-heating line, Wang et al. [5] established a numerical calculation model of multiple heating lines for the forming of sail plates and studied the interaction between heating lines.

Most of the researchers focus on the forming of single-curved plates and sail plates, and the material of the steel plate is mostly low-carbon steel. None of them have researched the forming of high-strength-steel saddle plates for ships. The current numerical calculation model in line heating is not suitable for saddle plates. A novelty of this paper is to study the numerical calculation of line heating for high-strength-steel saddle plates. As a result, the feasible numerical calculation model is established for saddle plate forming successfully. Following the temperature and deformation generated of the initial heating line and non-initial heating line are analyzed. The factors affecting the forming of high-strength-steel saddle plates are analyzed. The influence laws of geometric parameters and forming parameters on shrinkage and deflection are researched. The research can also provide ideas for research about plate forming in the fields of aerospace manufacturing, chemical pressure vessels and precision manufacturing.

## 2. Numerical Calculation of Line Heating for High-Strength-Steel Saddle Plates

**Figure 1:**
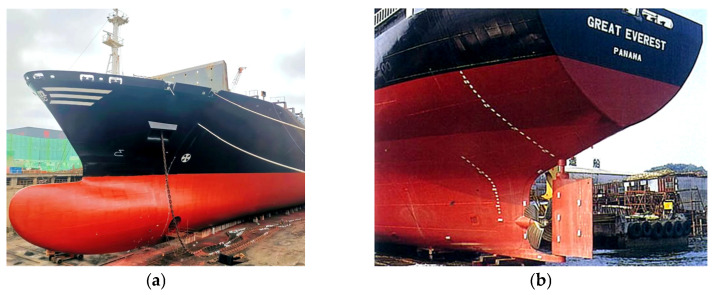
The location of complex shapes at the ship: (**a**) bow; (**b**) stern.

**Figure 2:**
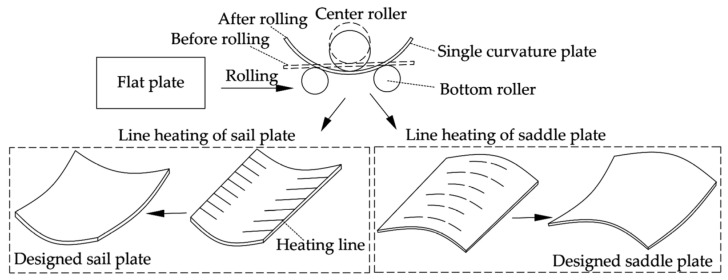
Forming process of sail plates and saddle plates.

Figure 1 shows the location of complex shapes at the bow and stern of ships, where double-curved plates are applied to meet the performance requirements of ship navigation. Sail plates and saddle plates are common double-curved plates in hull plates. Compared to sail plates, the forming quality of saddle plates is more important for improving the resistance performance of ships. Therefore, higher accuracy is required in the forming process of saddle plates. Figure 2 shows the forming process of sail plates and saddle plates. First, the flat plate is rolled into single-curved plates on the roller bed. Then, the double-curved plate is obtained by line heating. The forming parameters are determined by experience, including heating position, heating line length and the heating velocity. Finally, templates are used to check forming accuracy of plates. If the curved plate to be formed does not match the design surface, the curved plate needs reforming until the accuracy meets the test requirements.

A flame heat source is a common type of heat source in line heating. There are advantages of convenient operation and cost saving in a flame heat source. The propylene flame commonly used in shipyards is selected as the heat source type in this paper. Propylene has the advantages of low cost, slow combustion speed and stable chemical properties, which is more suitable for shipyard environments. The chemical equation of propylene combustion consists of primary combustion and secondary combustion.

(1) $$C_{3}H_{6}+4.5O_{2}=3{\mathrm{CO}}_{2}+3H_{2}O+2175\;\mathrm{kJ}/\mathrm{mol}$$

The equation of primary combustion reaction:

(2) $$C_{3}H_{6}+1.5O_{2}=3\mathrm{CO}+3H_{2}+391\;\mathrm{kJ}/\mathrm{mol}$$

The equation of secondary combustion reaction:

(3) $$\mathrm{CO}+3H_{2}+3O_{2}=3{\mathrm{CO}}_{2}+3H_{2}O+1784\;\mathrm{kJ}/\mathrm{mol}$$

The oxygen required for primary combustion reaction is supplied by a gas cylinder. The oxygen required for secondary combustion reaction is obtained from the air. The heat generated by the complete combustion of 1 mol propylene is 2175 kJ.

**Figure 3:**
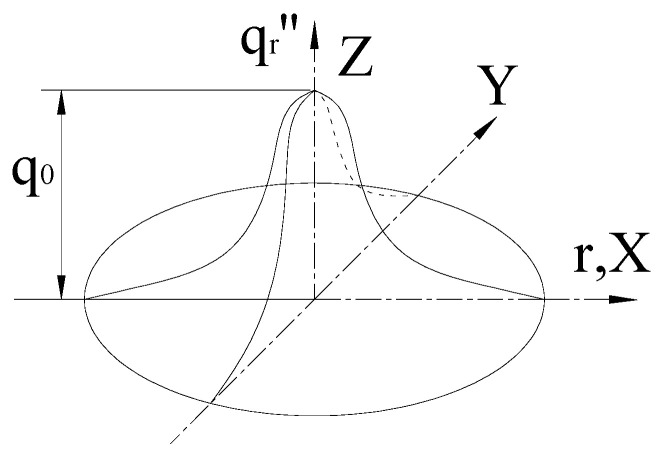
Gauss distribution model of heat flux.

The numerical calculation of line heating needs to select the suitable heat source model. Rykalin [22] believes that the distribution of heat flux along the radius direction of a gas jet is similar to the shape of a Gaussian distribution curve. Figure 3 shows the Gaussian distribution model of heat flux.

In polar coordinates, the heat flux $q^{\prime \prime }\left(r\right)$ at a certain distance from the heating center is expressed as follows:

(4) $$q^{\prime \prime }\left(r\right)=\frac{{3Q}_{C_{3}H_{6}}A\eta }{{\pi r}_{0}^{2}}\exp(-3{\left(r/r_{0}\right)}^{2})$$

where *A*, *η*, $r_{0}$, and *r* are the flow rate of propylene, thermal efficiency, effective radius of a heat source, and the distance from heating center, respectively.

**Figure 4:**
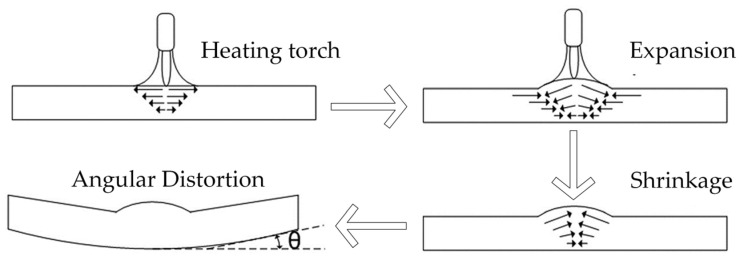
Mechanism of line heating.

Figure 4 shows the line heating mechanism. The line heating process consists of two stages: the heating stage and the cooling stage. In the heating stage, a heat source is applied to the upper surface of the plate, causing the it to expand. Subsequently, in the cooling stage, the surface temperature of the steel plate rapidly decreases as it exposed to cooling water, resulting in plate shrinkage. The degree shrinkage during the cooling stage exceeds the expansion that occurred in the heating stage. As a result, the plate undergoes irreversible plastic bending deformation. In summary, due to the temperature gradient across different regions of the plate, thermal expansion and contraction occur, resulting in the generation of internal stress and subsequent deformation of the steel plate.

**Figure 5:**
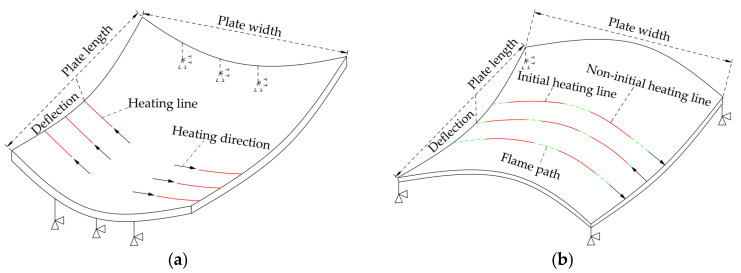
Forming characteristics of line heating for complex curved plates: (**a**) sail plates and (**b**) saddle plates.

**Figure 6:**
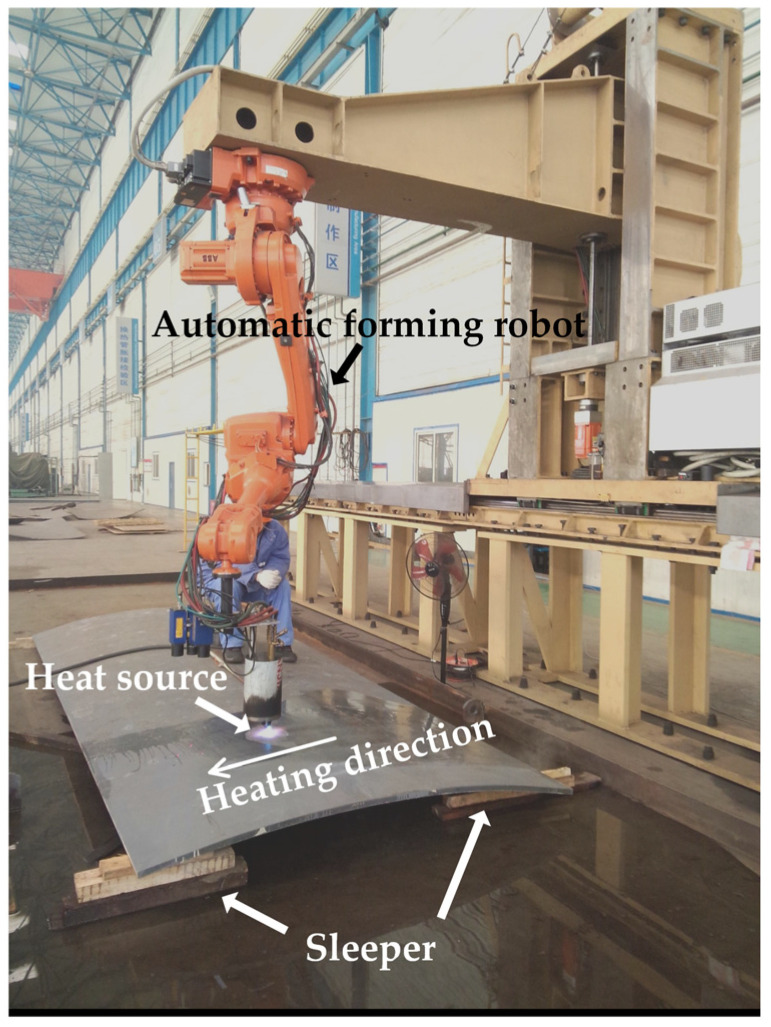
The actual Forming process of saddle plates.

There are different process characteristics in line heating of saddle plates and sail plates. Figure 5a shows the line heating of the sail plate. In actual processing process of sail plates, sleepers are padded on both ends of plate length direction. The constraint of sail plates can be equivalent to setting three unidirectional supports under the both ends of the plate. Figure 5b shows the line heating of the saddle plate. The actual forming process of saddle plates in shipyard is shown in Figure 6. There are four sleepers padded at the four corners of plates. The constraint of saddle plat can be equivalent to setting four unidirectional supports at the vertices of the plate lower surface. The heating lines are arranged in the middle of the flame path. The number of heating lines arranged on each flame path is not equal. The heating directions of adjacent flame path are opposite. The heating lines of saddle plates include initial heating line and non-initial heating line. In each flame path, the heating line that is first heated is called the initial heating line, the remaining heating lines are called non-initial heating line. The line heating process of saddle plates is more complicated than that of sail plates, so the existing research results of sail plates cannot be directly applied to saddle plate forming.

### 2.1. The Thermal Elastic–Plastic Theory of Line Heating

The line heating process involves a concentrated heat input, which causes stress and deformation of the plate. The calculation of stress and deformation is based on the analysis of the temperature field in the line heating process. The thermal elastic–plastic analysis of line heating in this paper makes the following assumptions: The material is an ideal plastic material and isotropic; the yield process of the material obeys the von Mises yield criterion; the material in plastic zone obeys flow rule; The material has no initial stress before being heated.

The time when the heat source reaches each position is different, and the mechanical properties of the material change with the change in temperature, which does not meet the conditions of total theory of plasticity. Therefore, the incremental theory is used to research the thermal elastic–plastic process of line heating. the thermal elastic–plastic process of line heating meets the stress–strain relationship and constitutive relations [23].

(1) The stress–strain relationship

The stress–strain relationship of the material in elastic or plastic state is as follows.

(5) $$\left\{d\varepsilon \right\}=\left[D\right]\left\{d\varepsilon \right\}-\left\{C\right\}dT$$

where $\left[D\right]$ is the elastic or elasto-plastic matrix and $\left\{C\right\}$ is a vector related to temperature.

In the elastic zone,

(6) $$\left\{D\right\}={\left\{D\right\}}_{e}$$

(7) $$\left\{C\right\}={\left\{C\right\}}_{e}={\left[D\right]}_{e}(\left\{\alpha \right\}+\frac{\partial {\left[D\right]}_{e}^{-1}}{\partial T}\left\{\sigma \right\})$$

where α is coefficient of linear expansion and T is temperature.

In the plastic zone, the material yield condition is set as follows,

(8) $$f\left(\sigma \right)=f_{0}\left(\varepsilon_{p},T\right)$$

where f is yield function and $f_{0}$ is function of yield stress related to temperature and plastic strain.

According to the flow rule, the plastic strain increment can be expressed as:

(9) $${\left\{d\varepsilon \right\}}_{p}=\lambda \{\frac{\partial f}{\partial \sigma }\}$$

(2) Constitutive relations

For a certain element of the structure, there is a constitutive relation as follows:

(10) $${\left\{dF\right\}}^{e}+{\left\{dR\right\}}^{e}={\left[K\right]}^{e}{\left\{d\delta \right\}}^{e}$$

where ${\left\{dF\right\}}^{e}$ is increment of element node force, ${\left\{dR\right\}}^{e}$ is the equivalent node increment of element initial strain caused by temperature, ${\left\{d\delta \right\}}^{e}$ is the increment of node displacement, and ${\left[K\right]}^{e}$ is the element stiffness matrix.

(11) $${\left[K\right]}^{e}=\int {\left[B\right]}^{T}\left[D\right]\left[B\right]dV$$

(12) $${\left\{dR\right\}}^{e}=\int {\left[B\right]}^{T}\left\{C\right\}dTdV$$

where $\left[B\right]$ is the matrix connecting element strain and the node displacement vector.

According to the element in the elastic zone or plastic zone, ${\left[D\right]}_{e},{\left\{C\right\}}_{e}$ or ${\left[D\right]}_{p},{\left\{C\right\}}_{ep}$ are used to replace $\left[D\right]$,$\left\{C\right\}$ to form the element stiffness matrix and equivalent node load. Then, the total stiffness matrix $\left[K\right]$ and the total load vector $\left\{dF\right\}$ are integrated to obtain the constitutive relations of the whole component as follows:

(13) $$\left[K\right]\left\{d\delta \right\}=\left\{dF\right\}$$

where $\left[K\right]=\sum {\left[K\right]}_{e},\left\{dF\right\}=\sum ({\left\{dF\right\}}^{e}+{\left\{dR\right\}}^{e})$.

Considering that the line heating process generally has no external force, the corresponding node force of the unit around each node is self-balanced, that is, $\sum {\left\{dF\right\}}^{e}=0$. Therefore, $\left\{dF\right\}=\sum {\left\{dR\right\}}^{e}$.

### 2.2. The Material Parameters of Low-Carbon Steel and High-Strength Steel

**Figure 7:**
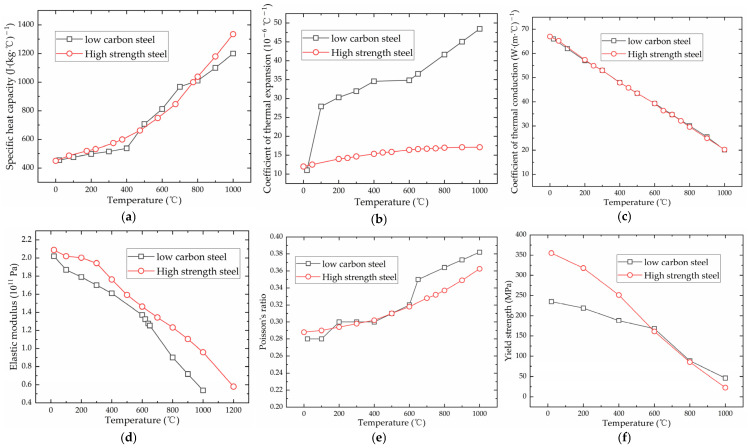
The material parameters of low-carbon steel and high-strength steel: (**a**) specific heat capacity; (**b**) coefficient of thermal expansion; (**c**) coefficient of thermal conduction; (**d**) elastic modulus; (**e**) Poisson’s ratio; (**f**) yield strength.

The material parameters for low-carbon steel and high-strength steel are obtained from [24,25]. Figure 7 displays the material parameters for both low-carbon steel and high-strength steel.

### 2.3. Reliability Verification of Numerical Calculation for Low-Carbon-Steel Saddle Plate

**Figure 8:**
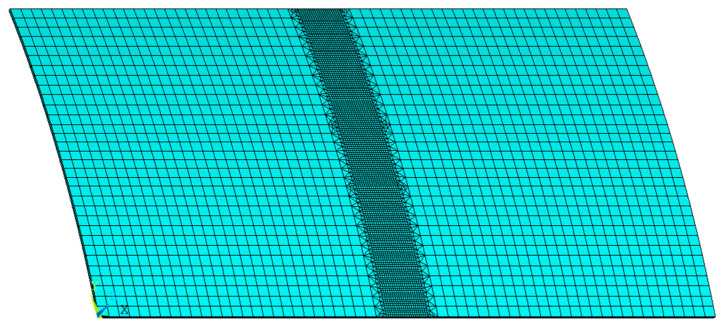
Finite element model of the line heating process.

**Figure 9:**
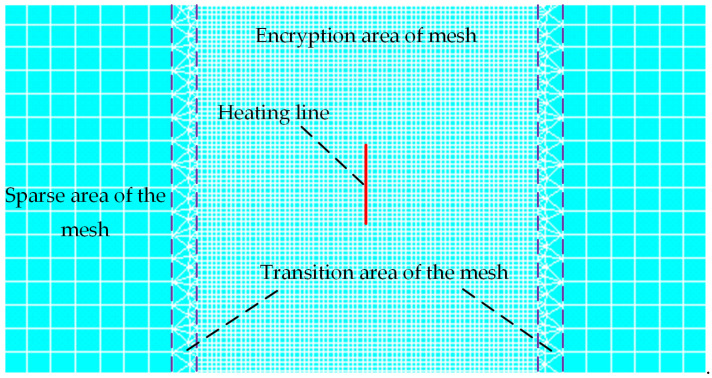
Mesh generation of the finite element model for saddle plate forming.

Based on the thermal elastic–plastic theory, the finite element simulation model of line hearting for saddle plates is established by software ANSYS 16.0. To study the influence of different geometric parameters and forming parameters on saddle plate deformation efficiently, the model is carried out by using Ansys Parametric Design Language (APDL) as shown in Figure 8. The mesh division of the finite element model for saddle plate forming is shown in Figure 9. In the process of line heating, the temperature gradient near the heating line is relatively high. The temperature gradient is relatively low at a certain distance from the heating line. Consequently, the mesh size decreases as the distance from the heating line decreases. The area within 0.1 m of the heating line is designated as encryption area, whose consists of hexahedral grid elements with a length of 0.01 m. The area more than 0.15 m away from the heating line is sparse area, which consists of hexahedral grid elements with a length of 0.05 m. The transition area is between the encryption and sparse area, which consists of triangular grid elements with a length of 0.02 m. SOLID186 elements are utilized for encryption area and sparse area of the mesh. SOLID185 elements are used for transition area of the mesh. Considering the process characteristics of the line heating for saddle plates, unidimensional LINK180 elements are set in the four vertices of the lower surface.

To verify the reliability of the numerical calculation for low-carbon-steel saddle plates, the plate numbered 8012-CPA1-5P on a 3900 TEU container ship is selected for the line heating experiment of saddle plates. The material of the actual hull plate is ordinary low-carbon steel. The geometric parameters measured of the hull plate are as follows. The plate length is 4.268 m. The plate width is 1.744 m. The plate thickness is 0.023 m. The radius of curvature is 3.5 m.

The material parameters, geometric parameters and forming parameters in numerical calculation are set according to real hull plate experiments. The heating line is positioned at the center of the plate, as shown in Figure 9. The heating line length is 0.35 m. The heating time is 310 s. By analyzing the temperature and deformation of numerical calculation and experiment for saddle plate forming, the accuracy of the numerical calculation model for low-carbon-steel saddle plates can be verified.

(1) Reliability verification of temperature

**Figure 10:**
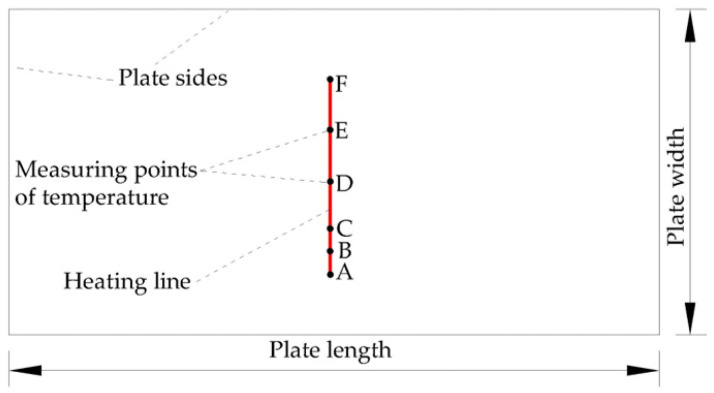
Distribution of measuring points for temperature.

**Figure 11:**
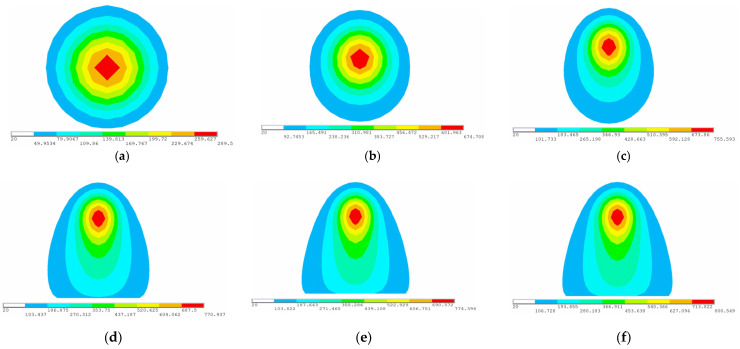
The temperature distribution at several moments (color scale units: °C): (**a**) time = 5 s, the heat source completes preheating at point A; (**b**) time = 42 s, the heat source moves to point B; (**c**) time = 83 s, the heat source moves to point C; (**d**) time = 161 s, the heat source moves to point D; (**e**) time = 239 s, the heat source moves to point E; (**f**) time = 315 s, the heat source moves to point F.

**Figure 12:**
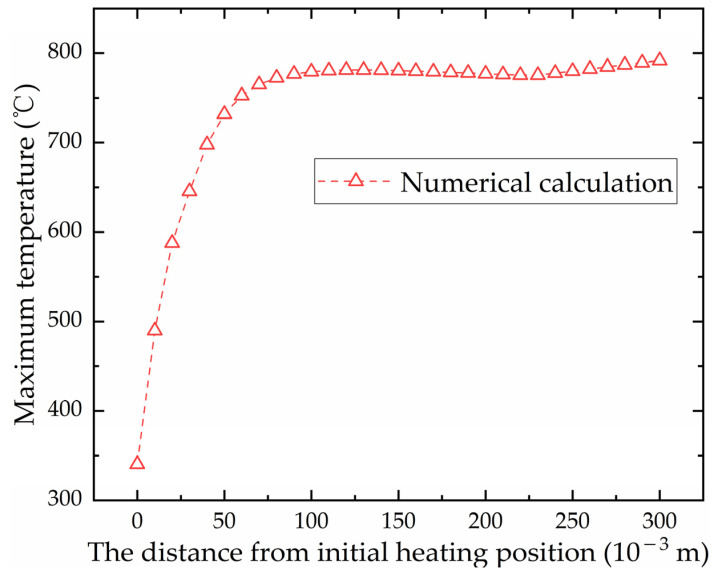
Maximum temperature in different positions along the heating line direction.

To observe the temperature field at different positions along the heating line, six points along the heating line are selected as measuring points of temperature. Figure 10 shows locations of the measuring points. Except for point B, the remaining five points are the quarter points of the heating line. Point A and Point F as measuring points can observe the temperature field at the completion of preheating and the end of heating. To carefully observe the changes in the temperature field during the early heating stage, point B, a midpoint between points A and C is selected as the measurement point. When the heat source reaches the measurement points of temperature, the temperature distribution at that time is shown in Figure 11. As shown in Figure 11a–c, the range of the temperature field is different and the maximum temperature varies greatly. As shown in Figure 11d–f, the range of the temperature field and the maximum temperature are similar. The temperature generated by the heat source increases first and then tends to stabilize with time. In order to further reveal the variation laws of temperature along the heating line, the maximum temperatures at different positions along the heating line in the numerical calculation are presented in Figure 12. The horizontal coordinate represents the distance from initial position of the heating line. From 0 to 0.075 m, the temperature of the plate surface rises rapidly. After 0.075 m, the temperature tends to be stable and maximum temperature is 800.549 °C.

**Figure 13:**
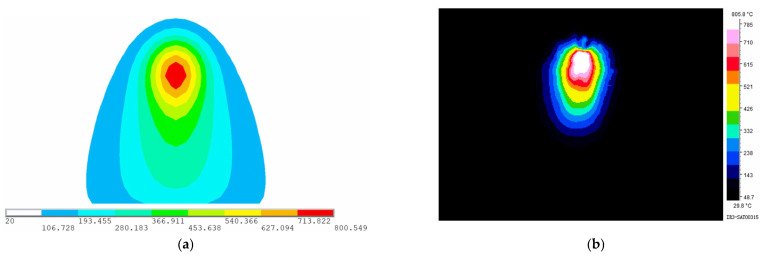
Surface temperature distribution of plates when the heat source reaches the end of heating line (color scale units: °C): (**a**) numerical calculation; (**b**) experimental measurement.

Figure 13 shows the temperature distribution in numerical calculation and experiment when the heat source reaches the end of the heating line. The temperature of experiment is measured by infrared thermal imager. The maximum temperature of the plate surface measured by numerical calculation and experiment is 800.549 °C and 805.8 °C. The accuracy of numerical calculation is high, indicating that this model can be applied to calculate the temperature of line heating for low-carbon-steel saddle plates.

(2) Reliability verification of shrinkage

**Figure 14:**
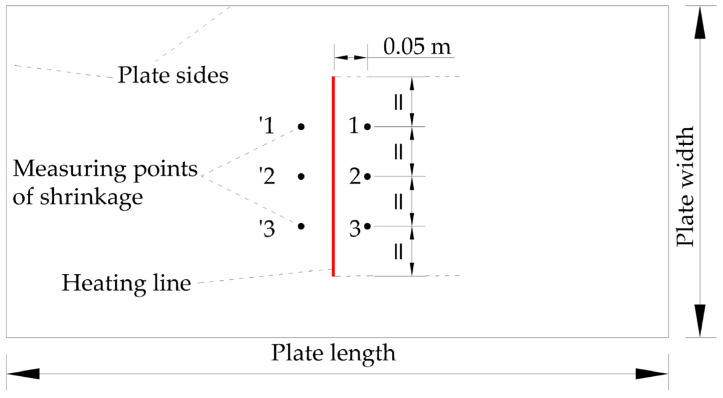
Distribution of measuring points for shrinkage.

The local deformation of line heating includes angular deformation and transverse deformation, which together cause the required deformation of the steel plate. It is difficult to analyze the proportion of angular deformation and transverse deformation to local deformation. The shrinkage in [26] is proposed instead of local deformation. Shrinkage occurs in the vicinity of the heating line during process for plate forming. To accurately measure the shrinkage, three pairs of measuring points are strategically placed 0.05 m away from the heating line on both sides, as depicted in Figure 14. These measuring points are clearly signed using maker pens. The distance between each pair of measure points is carefully measured using a measuring tool before and after the process operation. The shrinkage is the difference in distance between each pair of measurement points before and after forming.

**Figure 15:**
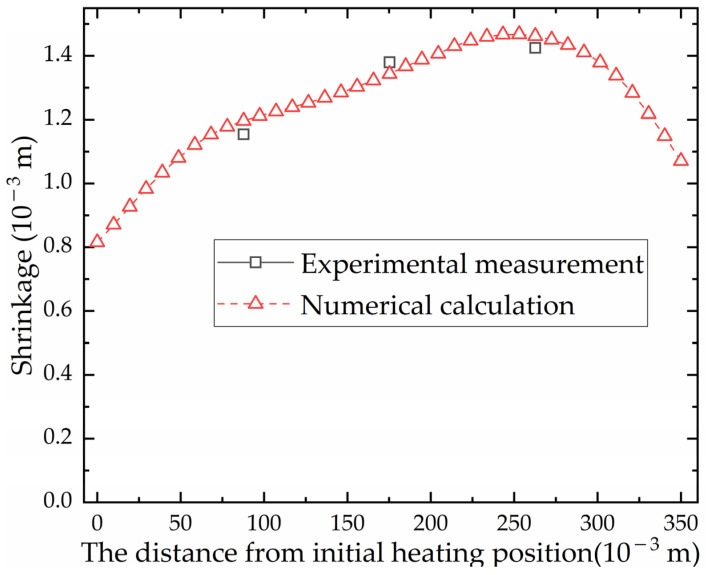
The shrinkage of low-carbon-steel saddle plates along the heating line direction in numerical calculation and experiment.

Figure 15 shows the shrinkage along the heating line direction of numerical calculation and experiment. The position with the maximum error between the numerical calculation and the experiment is 0.0877 m from the initial position. The maximum relative error is 3.58%, which is acceptable for engineering calculations. As the heat source moves, the shrinkage increases first and then decreases. The position of the maximum shrinkage is not the end point, but the position behind the middle of the heating line. Although the temperature at the end point is the highest, the area of the cold metal around it is large. The cold metal inhibits the expansion process of the heating stage, resulting in smaller shrinkage. The result shows that the numerical simulation model can be applied to calculate the shrinkage of line heating for low-carbon-steel saddle plates.

### 2.4. Feasibility Verification of Numerical Calculation for High-Strength-Steel Saddle Plates

The reliability of the numerical calculation model for low-carbon steel has been verified previously. Because the line heating experiment for high-strength-steel saddle plates has not been carried out, the feasibility of numerical calculation for high-strength steel is verified by comparing with the results of low-carbon steel.

The selected high-strength steel type is EH36. The geometric parameters of the finite element model for high-strength-steel and low-carbon-steel plates are same before heating. The geometric parameters are as follows. The plate length is 3.0 m. The plate width is 1.5 m. The plate thickness is 0.014 m. The radius of curvature is 5.0 m. The forming parameters of these two materials are same. The heating line is positioned at the center of the plate. The preheating time at the start point of heating line is 5 s. The heating line length is 0.30 m. The heating time is 171 s.

(1) Feasibility verification of temperature

**Figure 16:**
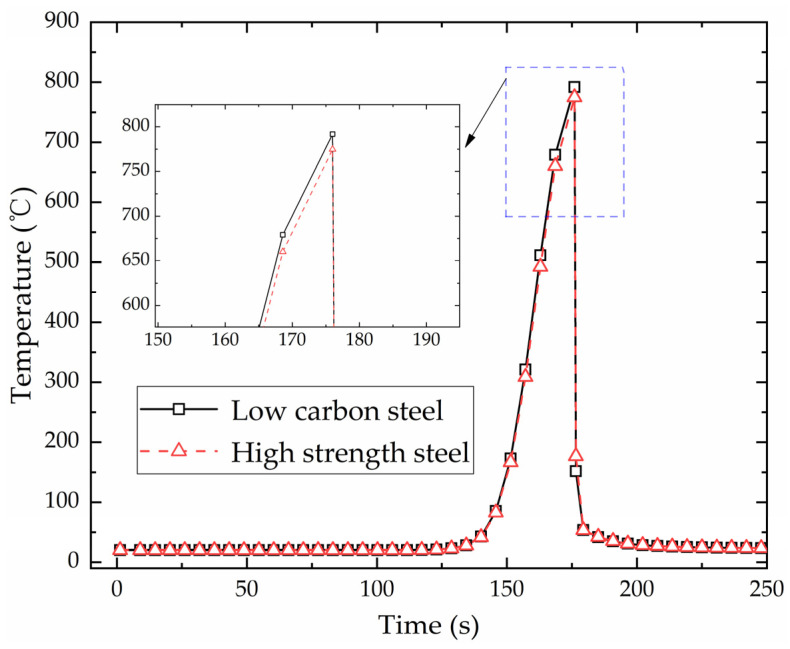
The temperature variation with time at the end of heating line for low-carbon steel and high-strength steel.

**Figure 17:**
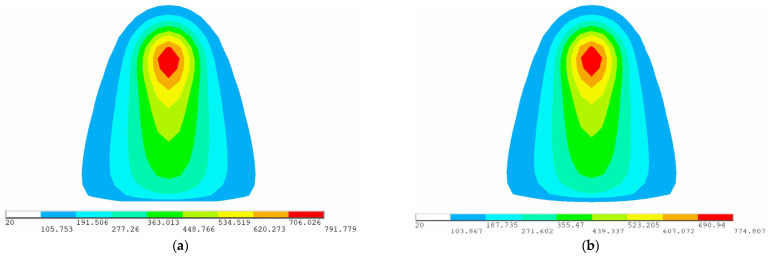
Surface temperature distribution of plates at the end of heating line (color scale units: °C): (**a**) low-carbon steel; (**b**) high-strength steel.

Figure 16 shows the temperature variation with time at the end of heating line for low-carbon steel and high-strength steel. The temperature variation of these two materials is consistent. The heat source is far from the end of heating line in the early stage of heating, which has no effect on the temperature at the end of heating line. After 125 s, the temperature increases with the heat source approaching. When the heat source reaches the end of heating line at 176 s, the temperature is the highest. Then, the temperature decreases rapidly due to the effect of cooling water. Figure 17 shows the temperature distribution of low-carbon steel and high-strength steel when the heat source reaches the end of heating line. The maximum temperatures of low-carbon steel and high-strength steel are 791.779 °C and 774.807 °C. The maximum temperature of these two materials has little difference. The distribution law of two materials in numerical simulation is same. High-temperature zones are concentrated in the center of the heat source, with similar temperature distribution lengths and widths. The result shows that the numerical calculation of temperature in high-strength-steel saddle plates is feasible.

(2) Feasibility verification of shrinkage and deflection

The plastic deformation of saddle plates in the process of line heating is expressed by shrinkage and deflection in this study. The end point of heating line is selected as the monitoring point of shrinkage and deflection. Deflection is the vertical displacement along the plate length direction, which is marked in Figure 5.

**Figure 18:**
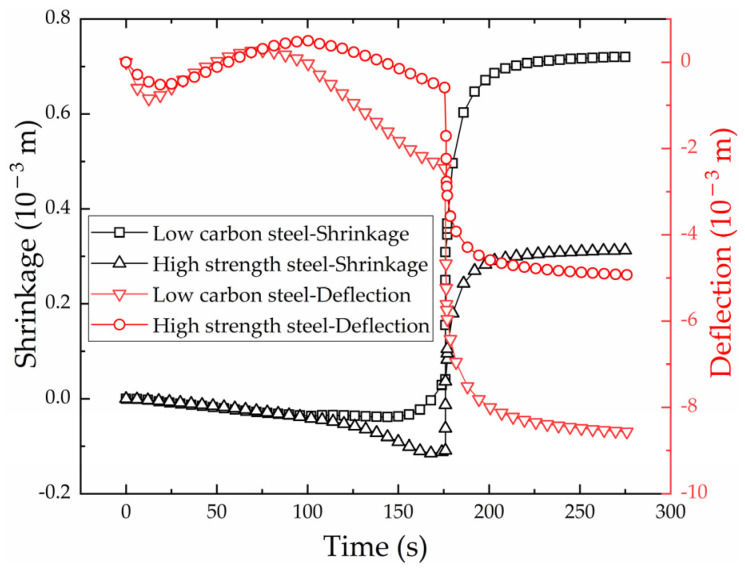
The variation of shrinkage and deflection with time for low-carbon steel and high-strength steel.

Figure 18 shows shrinkage variation of the monitoring point with time. The shrinkage curve of high-strength steel is the same as that of low-carbon steel. The shrinkage remains negative as the heat source approaches from 0 to 176 s. This is because the plate expands by heat, and the expansion becomes larger and larger as the temperature increases. The heat source reaches the end of heating line at 176 s, and the line heating process begins to enter the cooling stage. Under the action of cooling water, the plate forms shrinkage sharply. Because the shrinkage is greater than the expansion, the shrinkage finally becomes positive and tends to a fixed value. The final shrinkage of high-strength steel and low-carbon steel is $3.1\;\times \;10^{-4}$ m and $7.2\;\times \;10^{-4}$ m.

Figure 18 also shows the deflection variation with time. The deflection curve of high-strength steel is same as that of low-carbon steel. When the heat source reaches the end point at 176 s, the plate forms shrinkage sharply because of the cooling water. The final deflection of high-strength steel and low-carbon steel is $4.92\;\times \;10^{-3}$ m and $8.56\;\times \;10^{-3}$ m.

**Figure 19:**
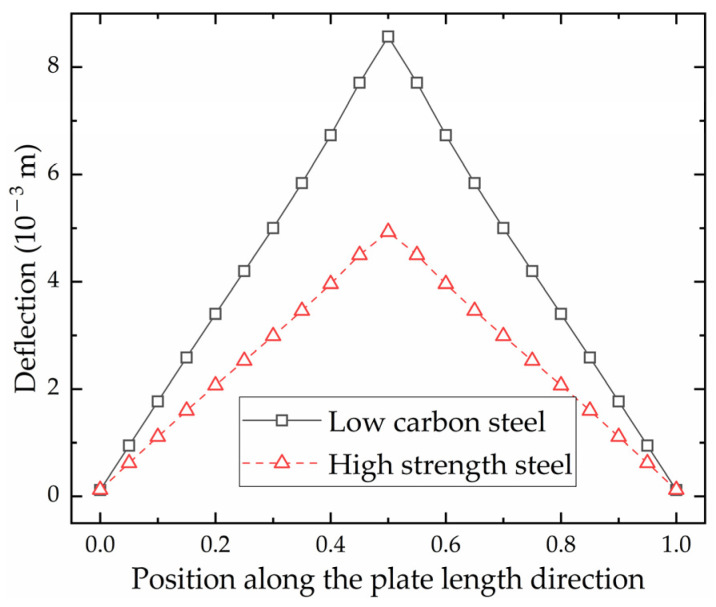
Deflection distribution of low-carbon steel and high-strength steel along plate length direction.

Figure 19 shows the deflection distribution along plate length direction of the section where the monitoring points are located. The horizontal coordinate is the ratio of the distance from the aft side to the plate length. The deflection distribution of high-strength steel is consistent with that of low-carbon steel. The closer to the heating line, the greater the deflection. This phenomenon is consistent with the laws of the line heating experiment for saddle plates in [27]. The shrinkage and deflection of high-strength steel are smaller than that of low-carbon steel under the same forming parameters. This difference can be attributed to the smaller coefficient of linear expansion exhibited by high-strength steel in comparison to low-carbon steel. The feasibility of utilizing numerical calculation to calculate shrinkage and deflection in line heating of high-strength-steel saddle plates is verified.

## 3. Numerical Calculation for High-Strength-Steel Saddle Plates with Multiple Heating Lines

### 3.1. Research on the Numerical Calculation Model with Multiple Heating Lines of High-Strength-Steel Saddle Plates

Considering the practical requirement for saddle plate forming, it is necessary to research the numerical calculation model with multiple heating lines of high-strength-steel saddle plates. The numerical calculation model with multiple heating lines of high-strength-steel saddle plates is established based on software ANSYS. The reasonable forming parameters such as heating position and heating line spacing are designed. The length of each heating line is 0.30 m. The heating time of each heating line is 125 s. The geometric parameters of the high-strength steel are as follows. The plate length is 3.0 m. The plate width is 1.5 m. The plate thickness is 0.014 m. The radius of curvature is 5.0 m.

**Figure 20:**
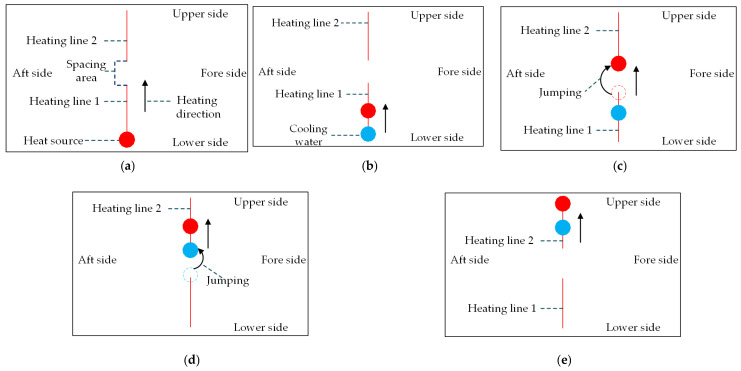
Forming process of high-strength-steel saddle plates with multiple heating lines: (**a**) preheating of steel plates; (**b**) the movement of the heat source and cooling water; (**c**) the heat source jumps to the starting point of heating line 2; (**d**) the cooling water jumps to the starting point of heating line 2; (**e**) heating line 2 is completely heated by the heat source.

Figure 20 shows the forming process for high-strength-steel saddle plates with multiple heating lines. The heating lines are arranged in the middle of plate length. The heating line 1 is initial heating line, and the heating line 2 is non-initial heating line. The initial position of heating line 1 is 0.40 m from the lower side. The initial position of heating line 2 is 0.70 m from the lower side. The spacing area is 0.10 m wide. The forming process is divided into five steps as follows. (a) The heat source stays at the starting point of line 1 for 5 s. (b) The heat source and the cooling water, with a constant distance of 0.15 m, move in the same direction at a predetermined velocity. (c) Once the heat source reaches the end of heating line 1, it transitions to the initial position of heating line 2 and continues its movement. (d) When the cooling water reaches the end of heating line 1, it jumps to the initial position of heating line 2 and continues its movement. (e) Once heating line 2 is completely heated by the heat source, the temperature of the whole plate rapidly decreases due to the cooling water.

**Figure 21:**
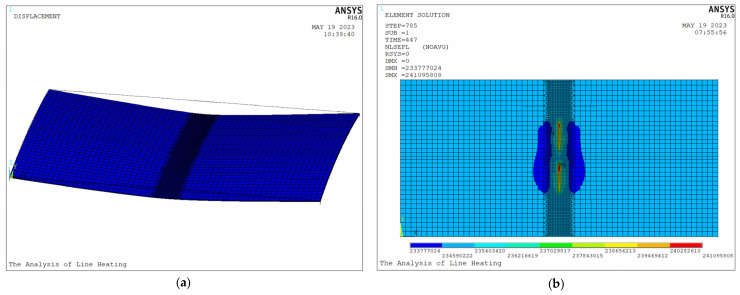
Distributions of (**a**) displacements and (**b**) plastic equivalent stress.

Figure 21 shows distributions of displacements and plastic equivalent stress after processing. As shown in Figure 21a, the final shape of the plate is saddle shape. The deflection in the middle of the plate is the largest, and the farther away from the plate, the smaller the deflection. As shown in Figure 21b, plastic equivalent stress is concentrated near the heating line.

**Figure 22:**
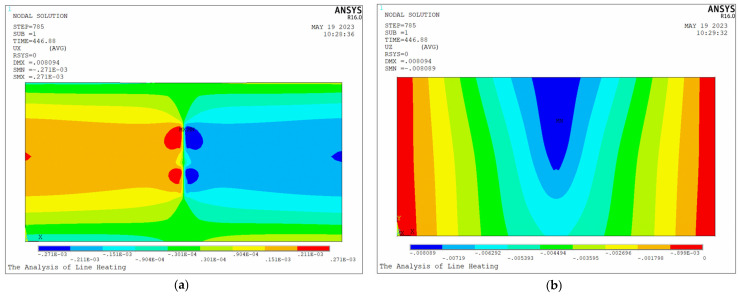
The (**a**) shrinkage and (**b**) deflection of the plate.

Figure 22 presents the shrinkage and deflection of the plate. Larger deformation occurs near the heating lines of the plate. The deformation field area produced by heating line 2 is larger than that produced by heating line 1. In order to study the differences between initial heating line and non-initial heating line, the following section will provide a detailed analysis of their effects on temperature and deformation.

### 3.2. Comparison of Initial Heating Line and Non-Initial Heating Line

(1) Comparison of temperature generated by the initial heating line and non-initial heating line

**Figure 23:**
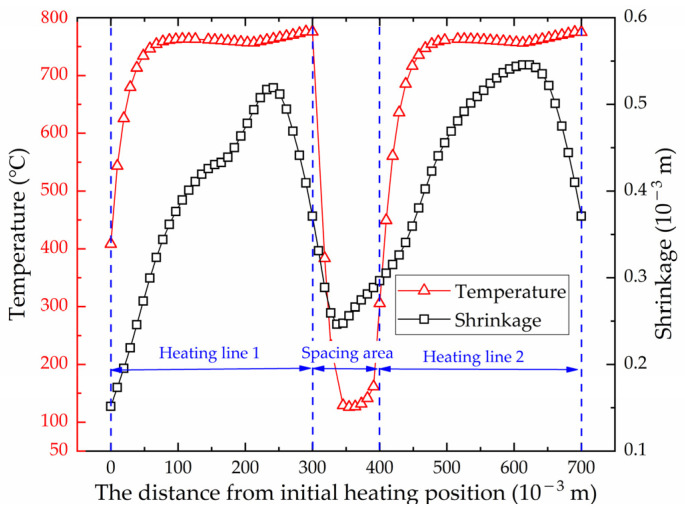
Distribution of maximum temperature and shrinkage along the heating line direction.

**Figure 24:**
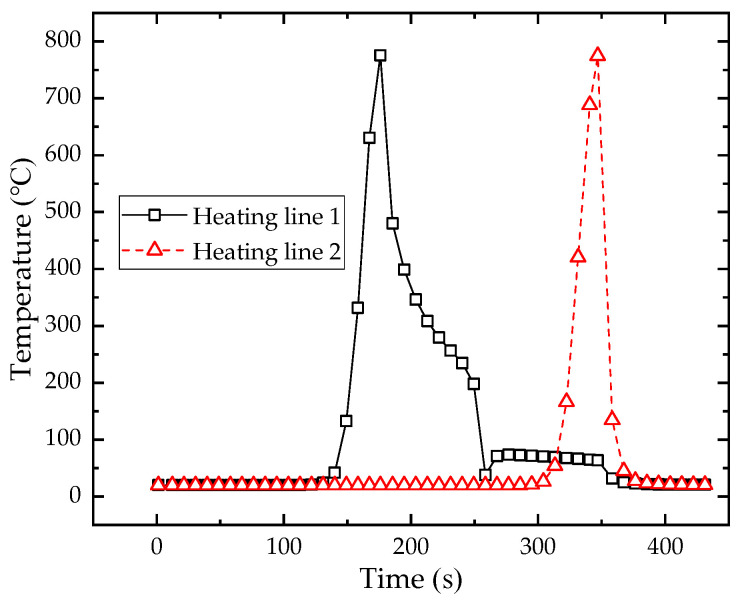
The temperature variation at the end of heating line 1 and heating line 2.

Figure 23 shows the maximum temperature distribution along the heating line in the numerical calculation model with multiple heating lines. The position of heating line 1 is 0 to 0.30 m and position of heating line 2 is 0.40 to 0.70 m. It can be seen that the temperature distribution laws of these two heating lines are similar. There is a preheating process at the initial position of heating line 1, so that the maximum temperature at the initial position of heating line 1 is higher than that of heating line 2. Figure 24 shows the temperature variation at the end of heating line 1 and heating line 2. The heating trend of these two heating lines is consistent. The temperature gradually increases with the approaching of the heat source. The maximum temperatures of heating line 1 and heating line 2 are 775.56 °C and 774.91 °C, with a difference of 0.65 °C. There are differences in the cooling stage of two heating lines. The heat source jumps directly to the initial position of heating line 2 when it reaches the end point of heating line 1. The cooling water is exerted to the entire plate surface when the heat source reaches the end of the heating line 2, which causes the cooling rate of heating line 2 is faster than that of heating line 1. When the heat source moves in heating line 2, part of the heat is transferred to the heating line 1, which leads to a slight increase in the temperature at the end of the heating line 1 at 255 s. The cooling water causes the plate temperature decreases rapidly when the heating stage is completed at 347 s. The temperature of these two heating lines is finally the same as the room temperature.

(2) Comparison of deformation generated by the initial heating line and non-initial heating line

Figure 23 shows the shrinkage at different positions along the heating line. The distribution trend of shrinkage generated by these two heating lines is similar. From the overall distribution, the shrinkage of heating line 2 is greater than that of heating line 1. The maximum shrinkage of heating line 1 and heating line 2 is $5.19\;\times \;10^{-4}$ m and $5.45\;\times \;10^{-4}$ m.

**Figure 25:**
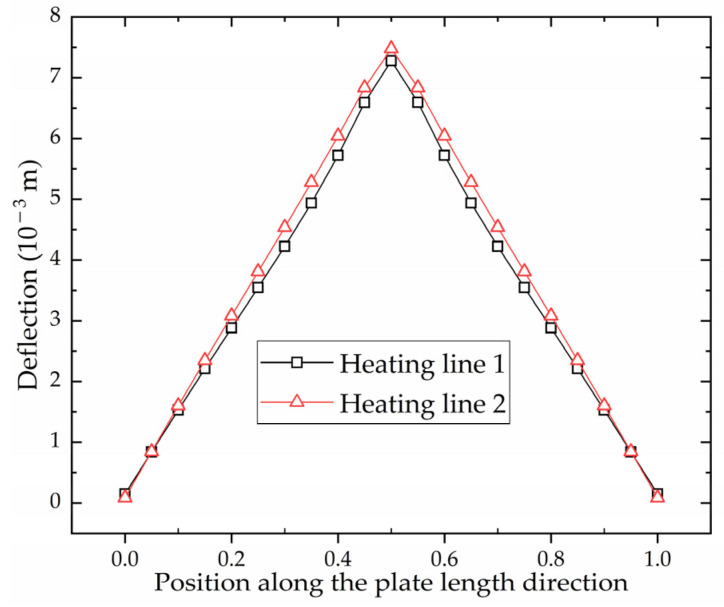
Deflection distribution along plate length direction of the section at the end of heating line 1 and heating line 2.

Figure 25 shows the deflection distribution along plate length direction of the section at the end of heating line 1 and heating line 2. The deflection distribution trend of both sections is the same. The maximum deflection is at the middle of the plate length. The maximum deflection of heating line 1 and heating line 2 is $7.28\;\times \;10^{-3}$ m and $7.48\;\times \;10^{-3}$ m. The deflection at any position generated by heating line 2 is greater than that of heating line 1. Figure 23 and Figure 25 show that the deformation caused by non-initial heating line is greater than that caused by initial heating line.

## 4. Influence Laws of Line Heating for High-Strength-Steel Saddle Plates

### 4.1. Analysis of Influencing Factors on the Line Heating of High-Strength-Steel Saddle Plates

**Figure 26:**
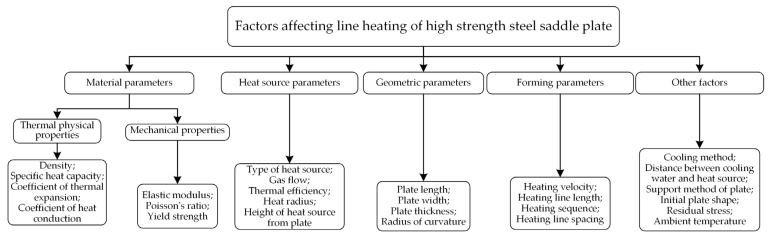
Factors affecting line heating of high-strength-steel saddle plates.

As shown in Figure 26, there are many factors affecting the deformation of line heating for high-strength-steel saddle plates. According to different types, the factors can be divided into material parameters, heat source parameters, geometric parameters, forming parameters and other influencing factors. Based on the actual forming process in a shipyard, the line heating of EH36 marine high-strength steel is studied. The material parameters of high strength have been determined. Propylene heat sources are selected to heat the plate, which has a good penetration and low cost. The heat source parameters are fixed according to the actual setting. The cooling method is tracking water cooling. The distance between cooling water and the heat source is 0.15 m. The ambient temperature is 20 °C. The plate is constrained by simply supported supports at four corners. The effects of heating line spacing, initial plate shape, and residual stress on the calculation results are not considered in this paper. The following only studies the influence of geometric parameters and forming parameters on the line heating of high-strength-steel saddle plates.

### 4.2. Effect of Geometric Parameters on Deformation of High-Strength-Steel Saddle Plates

**Figure 27:**
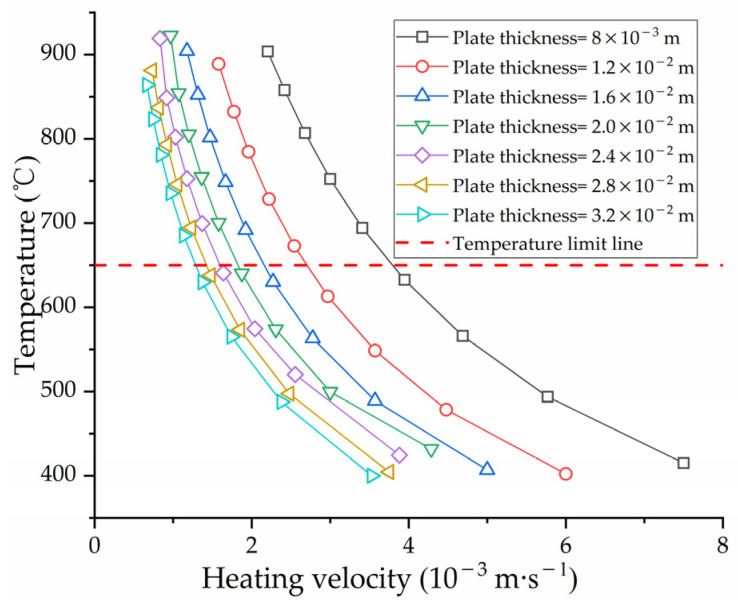
Effect of heating velocity on maximum temperature with different thickness.

According to the field experience of line heating, the heating velocity is closely related to the temperature of the plate surface, which affects the size of the deformation. To obtain a larger deformation, a slower heating velocity is usually required. Figure 27 shows the effect of heating velocity on maximum temperature of plate surface with different plate thicknesses. Under the same plate thickness, the slower the heating velocity, the higher the maximum temperature of plate surface. At the same heating velocity, the greater the plate thickness, the larger the cold metal area in the thickness direction. The heat will be absorbed by the cold metal, which leads to a decrease in the surface temperature.

**Figure 28:**
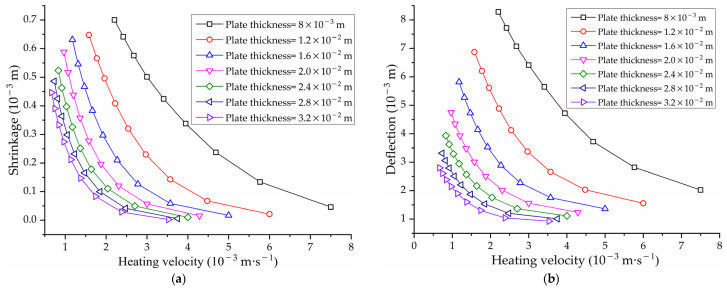
Effect of heating velocity on shrinkage and deflection with different thickness: (**a**) effect of heating velocity on shrinkage; (**b**) effect of heating velocity on deflection.

To obtain the influence laws of shrinkage and deflection, the midpoint and endpoint of the heating line are monitoring points for shrinkage and deflection, respectively. Figure 28 shows the effect of heating velocity on shrinkage and deflection under different plate thicknesses. It is beneficial for the forming of the saddle plate to reducing the heating velocity. However, the material performance of the steel plate will be decreased when the surface temperature is too high. According to the Chinese shipbuilding quality standard [28], the maximum surface temperature of EH36 marine steel with carbon equivalent greater than 0.38% should be lower than 650 °C when cooled immediately after heating. The temperature limit line is shown as the dotted line in Figure 27. Under the premise that the plate material is not damaged, the heating velocity corresponding to the temperature limit line is the velocity that causes the maximum deformation of the saddle plate.

To explore the effect of geometric parameters on the deformation of high-strength-steel saddle plates, it is necessary to ensure the same forming parameters. The heating line is arranged in the middle of plates. The heating velocity is obtained from the temperature limit line. According to the actual hull plate size at the current stage of the shipyard, the geometric parameters are set as follows. The plate thickness ranges from $8.0\;\times \;10^{-3}$ to $3.2\;\times \;10^{-2}$ m. The plate length ranges from 1.5 to 3.5 m. The plate width ranges from 1.0 to 2.5 m. The radius of curvature is from 1.0 to 5.0 m. The influence of geometric parameters such as plate thickness, plate length, plate width and radius of curvature on the line heating of saddle plates is analyzed.

(1) Effect of plate thickness on shrinkage and deflection of high-strength-steel saddle plates

Figure 28 shows the effect of heating velocity on shrinkage and deflection under different plate thicknesses. Under the same heating velocity, the greater the plate thicknesses, the smaller the shrinkage and deflection. This is because the thicker plate requires more energy to deform. For thicker plates, a smaller heating rate is required to achieve deformation.

(2) Effect of plate length on shrinkage and deflection of high-strength-steel saddle plates

**Figure 29:**
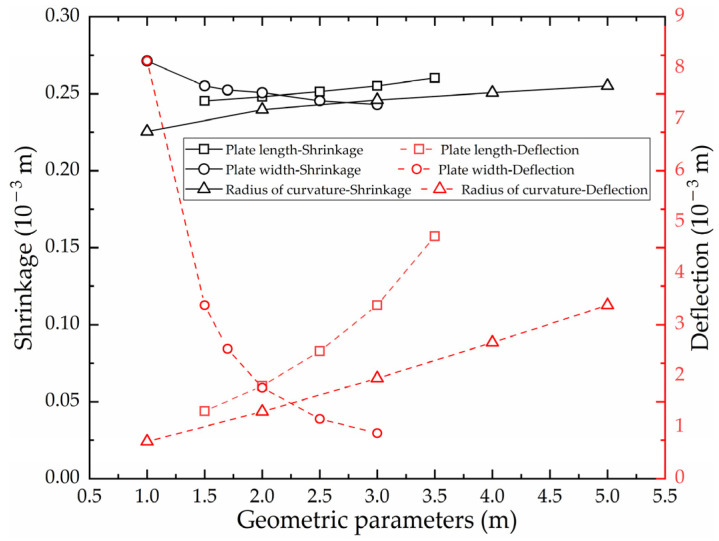
Effect of geometric parameters on shrinkage and deflection.

As shown in Figure 29, with other factors keep constant, the effect of plate length on shrinkage is little, but the effect on deflection is obvious. The larger the plate length of high-strength steel, the greater the deflection of the saddle plate.

(3) Effect of plate width on shrinkage and deflection of high-strength-steel saddle plates

As shown in Figure 29, with other factors keep constant, the effect of plate width on shrinkage is little, but the effect on deflection is obvious. The larger the plate width of high-strength steel, the smaller the deflection of the saddle plate.

(4) Effect of radius of curvature on shrinkage and deflection of high-strength-steel saddle plates

As shown in Figure 29, with other factors keep constant, the influence of radius of curvature on shrinkage is little, but the influence on deflection is obvious. The larger the radius of curvature of high-strength steel, the greater the deflection of the saddle plate.

### 4.3. Effect of Forming Parameters on Deformation of High-Strength-Steel Saddle Plates

To research the effect of forming parameters on the deformation of high-strength-steel saddle plates, the geometric parameters of the steel plate are set to fixed values as follows. The plate length is 3.0 m. The plate width is 1.5 m. The plate thickness is 0.014 m. The radius of curvature is 5.0 m.

(1) Effect of heating velocity on shrinkage and deflection of high-strength-steel saddle plates

Figure 28 shows the effect of heating velocity on the shrinkage and deflection of high-strength-steel saddle plates. The faster the heating velocity, the smaller the shrinkage and deflection generated. This law is consistent with the result that heating velocity is inversely proportional to the deformation in [27]. This is caused by the low temperature of the plate surface. It is beneficial for the forming of the saddle plate to reducing the heating velocity. To deform the plate, it is necessary to reduce the heating velocity. However, if the heating velocity is too low, the surface temperature of the steel plate will be too high, which will lead to the reduction in the performance of the steel plate and even the destruction of the material. Too low heating velocity could cause the temperature to be too high, which will lead to reduced performance and even material damage of the steel plate. The heating velocity should be controlled reasonably in line heating.

(2) Effect of heating line length on shrinkage and deflection of high-strength-steel saddle plates

**Figure 30:**
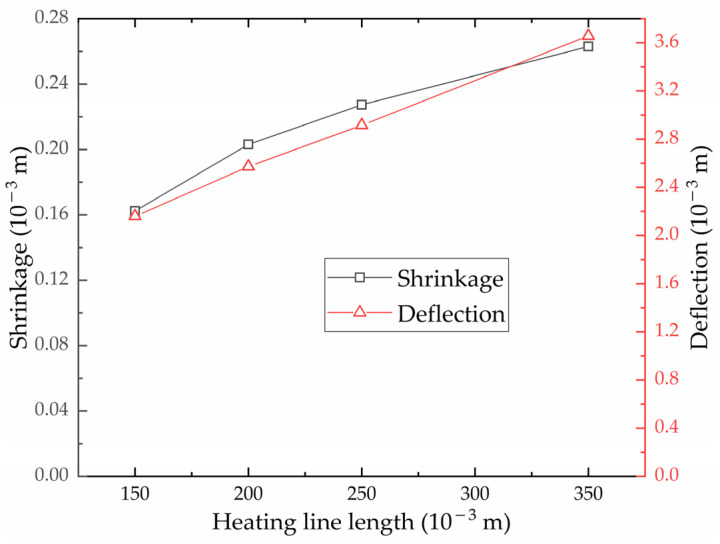
Effect of heating line length on shrinkage and deflection.

Figure 30 shows the effect of heating line length on the shrinkage and deflection of high-strength-steel saddle plates. The larger the heating line length, the greater the shrinkage and deflection generated. This is because the larger the heating line length is, the more the heating time is, and the more heat the steel plate absorbs. Due to the limitation of plate width, the heating line length cannot be arbitrarily set. The deformation to be processed and plate width should be considered when the heating line length is designed.

(3) Effect of the heating sequence on shrinkage and deflection of high-strength-steel saddle plates

The effect of the heating sequence on the deformation of the saddle plate is reflected in the fact that the heating sequence affects the position of the initial heating line and the non-initial heating line. Figure 23 and Figure 25 show the effect of the heating sequence on the shrinkage and deflection of high-strength-steel saddle plates. The shrinkage and deflection caused by non-initial heating line is greater than that caused by initial heating line.

## 5. Conclusions

A numerical calculation model of multiple heating lines for high-strength-steel saddle plates was established in this paper. The influence laws of geometric parameters and forming parameters on plate forming for high-strength-steel saddle plates were researched. By utilizing these influence laws, deformation can be predicted according to geometric parameters and forming parameters. The research can provide valuable references into the prediction for forming parameters of high-strength-steel saddle plates. The conclusions of this study are summarized as follows.

1. Based on ANSYS APDL, a numerical calculation model for saddle plates in line heating is established successfully. The accuracy of the numerical calculation model for low-carbon steel has been verified by comparing the results experimental results obtained from a real hull plate, with a relative error of less than 4%. The temperature distribution of high-strength steel is similar to that of low-carbon steel. The variation of shrinkage and deflection for high-strength steel is similar to that for low-carbon steel. The feasibility of numerical calculation for high-strength-steel saddle plates was verified.
2. The numerical calculation model with multiple heating lines of high-strength-steel saddle plates is established. The model can be used to analyze the temperature distribution and deformation distribution of initial heating line and non-initial heating line. The temperature distribution of the initial heating line and the non-initial heating line is similar. The maximum temperature difference between initial heating line and non-initial heating line is 0.65 °C. The deformation induced by non-initial heating line is approximately 5% greater than that of initial heating line.
3. For the effect of geometric parameters on the line heating of high-strength-steel saddle plates, the larger the plate thickness, the smaller the shrinkage and deflection. Plate length, plate width and curvature radius have little effect on shrinkage. The deflection is positively correlated with plate length and radius of curvature. The deflection is negatively correlated with plate width. As for deflection, there is a positive correlation with plate length and curvature radius, and plate width exhibits a negative correlation with deflection.
4. Regarding the effect of forming parameters on the line heating of high-strength-steel saddle plates, the slower the heating velocity, the smaller the shrinkage and deflection. The larger the heating line length, the greater the shrinkage and deflection.

The numerical calculation model in the can calculate the determination of the deflection of high-strength-steel saddle plates based on geometric and forming parameters. The prediction of forming process parameters has not been studied in this research. The influence laws of deformation in this study can lay a foundation for the prediction of forming parameters for high-strength-steel saddle plates.

## Data Availability

Not applicable.
